# Unrevealing the interaction between O_2_ molecules and poly(3-hexylthiophene-2,5-diyl) (P3HT)†

**DOI:** 10.1039/d2ra02969c

**Published:** 2022-06-23

**Authors:** Marcelo Fernandes, Ernesto Osvaldo Wrasse, Caio Junji Kawata Koyama, Florian Steffen Günther, Douglas José Coutinho

**Affiliations:** Federal University of Technology – Paraná (UTFPR) 85902-490 Toledo-PR Brazil douglascoutinho@utfpr.edu.br; São Carlos Institute of Physics, University of São Paulo P. O. Box 369 13560-970 São Carlos-SP Brazil f.guenther@ifsc.usp.br

## Abstract

Stability of π-conjugated organic materials remains a critical issue for applications in which these materials and devices based on them are exposed to ambient conditions. Particularly, the initial steps of the reversible and irreversible degradation by molecular oxygen exposure are still not fully explored. Here we present a theoretical study using density functional theory (DFT) to investigate the oxygen effects on the electronic properties of poly(3-hexylthiophene-2,5-diyl) (P3HT). Our results show that trap-states are introduced in the energy gap between the highest occupied and the lowest unoccupied molecular orbitals by the O_2_ molecule and both singlet and triplet states can be formed irrespectively of the existence of chain defects. A crossing between the potential energy surfaces of singlet and triplet states was observed for smaller distances of the oxygen molecule to the nearest thiophene ring, which was identified as being the first step towards irreversible degradation.

## Abstract 2

Stability of π-conjugated organic materials remains a critical issue for applications in which these materials and devices based on them are exposed to ambient conditions.

## Graphical abstract

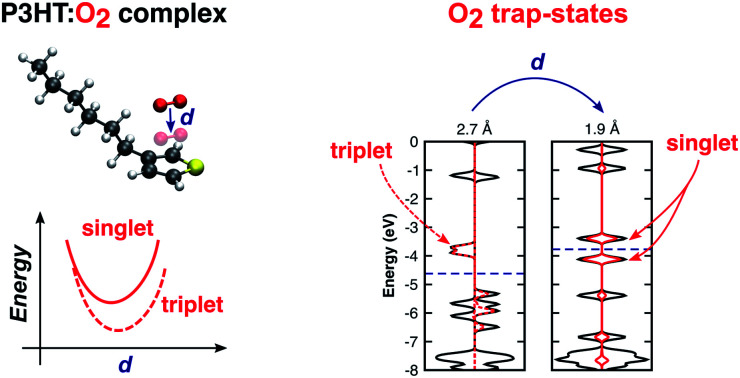

## Introduction

Organic electronic materials based on polymers and small molecules have shown a wide-range of technological applications due to their relevant electrical, optical, mechanical and biocompatible properties.^1^ Practical demonstrations based on these characteristics include: organic light-emitting diodes (OLEDs) for displays and lighting,^2^ organic field-effect transistors (OFETs) for a new generation of computing electronics,^3^ organic photovoltaics (OPVs) for light harvesting,^4,5^ and sensors, especially for bio-applications.^6,7^ The mechanical and optoelectronic properties of organic electronic materials as well as sensors and devices composed of them, however, are not only determined by the molecular structure but also by other factors such as doping, film morphology, or impurity-related defects.^8–11^ Those aspects do typically not depend on the synthesis but on the processing conditions and the environment in which the devices operate. It is therefore important to remark that some modification of the mechanical or optoelectronic properties of organic electronic devices occur non-intentionally, *e.g.* as a result of oxygen and moisture diffusion during material processing and/or during device operation.^12–18^

The interactions between oxygen and conjugated polymers can be divided in two categories according to their reversibility. Irreversible degradation typically involves a chemical reaction of O_2_ with the conjugating polymeric chain, resulting in the formation of new covalent bonds between oxygen and (but not restricted to) carbon.^19,20^ In the most severe cases, this leads to chain scission, *i.e.* a drastically reduced conjugation, and consequently in a reduction of the absorption band and in poor conductivity. This process might also be catalysed when organic materials are exposed to air (oxygen) and light simultaneously, in a process known as photo-oxidation.^20,21^ Reversible effects, on the other hand, originate from a physical interaction (non-covalent) between the polymer chain and the oxygen molecule, giving rise to the formation of a charge–transfer complex (CTC). This greatly increases the free hole concentration (p-doping) where the positive charges in the polymer are compensated by negative counter-charges formed by immobile superoxide anions.^16,22–24^ The O_2_ adsorption/desorption process and, consequently, the doping and de-doping of conjugated polymers, typically occurs in the time interval from minutes to hours and is strongly dependent on the ambient conditions such as oxygen pressure, light and temperature.^25^ Consequences of higher oxygen concentration on OFET response include: increase of carrier density due to induced p-type doping,^25^ but decrease of charge mobility,^23^ shift of the threshold-voltage (up to +20 V) and increase in the off-current by several orders of magnitude.^26^ In organic photovoltaic devices (OPVs), the exposure to oxygen results in losses of fill factor (FF) and reduced open-circuit voltage (*V*_OC_).^27,28^ Seemann *et al.* have also observed that the mobile holes form a space charge region near the electrodes, shielding the electric field inside the photoactive layer and hence hampers charge carrier extraction, which leads to the observed loss in short-circuit current (*J*_SC_).^29^

While irreversible degradation is a mark of the total death of the original optoelectronic properties, a partial or even a full recovery from reversible degradation is possible. Nonetheless, the reversibility might take as long as weeks under vacuum and room temperature, but can be shortened to minutes when heated up to the polymer's glass transition.^25,29^ Therefore, although the dynamics of the oxygen doping process is a key question from both fundamental and practical points of view, it has mostly been studied experimentally and discussed phenomenologically. Theoretical studies, however, have most focused on the irreversible chemical interactions.^19,21,30,31^ We therefore aim at closing this gap and at getting a better understanding of how the electronic structure of conjugated polymers change in the proximity of O_2_ molecules. For this, we restrict our studies to the organic conjugated semiconductor poly(3-hexylthiophene-2,5-diyl) (P3HT), which has been one of the most extensively studied materials in the research field of organic electronics and is still the material of choice for many novel analyses and characterisation techniques.

In this work, we study the interaction between oxygen and P3HT monomer/oligomers using unrestricted density functional theory (DFT) calculations. Our results show that the triplet nature of O_2_ is the origin of in-gap trap-states that cause reversible degradation. We found that for closer proximity the triplet characteristic vanishes and hybridised frontier orbitals with contributions of oxygen as well as the closest unit of P3HT are formed. We characterise this as initiation of the formation of new covalent bonds, *i.e.* irreversible degradation. These observations were made for P3HT monomer as well as for oligomers models in ordered and disordered orientations. This shows the generality of our conclusions. Even though we restricted our study to P3HT, we believe that the herein reported observations also hold for other materials since many semiconducting polymers involve thiophene derivatives.

## Computational details

To describe the interaction between P3HT and molecular oxygen, quantum chemical calculations within the framework of DFT as implemented in the SIESTA^32,33^ code were performed. In this approach the electronic charge density for the ground state is obtained through the Kohn–Sham orbitals, which are expanded in a linear combination of atomic orbitals. Moreover, a double-zeta basis with polarisation functions was employed. A good description of the ground state electronic density was achieved by using an equivalent energy cut-off of 170 Ry for grid integration. As the valence electrons are more effectively involved in the physical and chemical bonds when compared to the inner electrons, only the former was included in the self-consistent field employed to solve the Kohn–Sham equations. The inner electrons and the nucleus are combined to form the ionic core, and the interaction between valence electrons and ionic cores is described by norm-conserving pseudopotentials proposed by Troullier–Martins.^34^ The exchange–correlation term was described by the local spin density approximation (LSDA) functional of Ceperley and Alder^35^ with the parametrization of Perdew and Zunger.^36^ LSDA has been demonstrated to be suitable to describe the electronic properties of a wide range of materials, including systems for which van der Waals interactions are important.^37–40^ To improve the LSDA results, the van der Waals interactions were included by the Grimme method.^41^ For evaluating the stability of the models proposed, the formation energy was calculated as the difference between the total energy of the P3HT:O_2_ complexes and the total energies of non-interacting oxygen and polymer were computed. Due to the triplet ground state of molecular oxygen, all calculations were performed at the unrestricted level. The P3HT polymer was modelled as a monomer as well as an oligomer with up to 13 repeat units terminated with hydrogen. For the latter, different defect geometries were considered including different positions of the alkyl-chain as well as torsions of thiophene rings around the bond to adjacent units. Apart from that, no further simplification was done. In particular, the hexyl side-chains have been considered explicitly.

The following strategy was adopted for describing the possible intermolecular orientations: (i) the isolated P3HT and O_2_ models were generated and optimised, (ii) P3HT:O_2_ complexes were generated by placing the oxygen on top of the polymer at different positions followed by an optimisation, and (iii) modifying the obtained equilibrium orientations and performing single-point calculations. To analyse the electronic structure, we consider the density of states (DOS) which we obtained by placing Gaussian curves with width 0.05 eV at the position of the obtained orbital energies. To study the (de-) localisation of the orbitals, we furthermore considered the partial DOS (pDOS) by weighing the individual Gaussians by the gross orbital populations.

## Results and discussion

### 3-Hexylthiophene monomer (3HT)

**Fig. 1:**
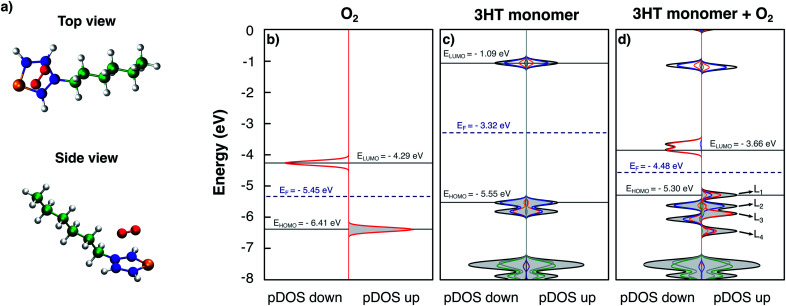
Top and side view of the equilibrium geometry of the 3HT:O_2_ system shown in a). DOS and pDOS of an isolated O_2_ molecule b), an isolated 3HT-monomer c) and the 3HT:O_2_ system d). Colours refer to the atoms as they are colour coded in a): oxygen – red, sulphur – orange, thiophene carbon – blue, alkyl carbon – green, and hydrogen – grey. Occupied states are indicated by grey shading.

At first, the simplest model of P3HT is considered: an isolated thiophene ring with the hexyl side-chain. Fig. 1a shows a top and a side view of the model in the obtained equilibrium geometry. The oxygen molecule lies parallel to the thiophene plane at a distance of 2.7 Å, but not symmetric with respect to the sulphur atom; possibly being also strongly influenced by the alkyl side-chain. Fig. 1 furthermore presents the obtained DOS and pDOS of the isolated oxygen molecule (Fig. 1b), the pristine 3HT monomer (Fig. 1c) and the 3HT:O_2_ complex (Fig. 1d), respectively. In Fig. 1b, the spin-down and spin-up DOS with peaks at −4.29 eV and −6.41 eV is shown, respectively, each formed from degenerated states: the two antibonding π_x_* and π_y_* orbitals. The splitting between spin-up and spin-down is a consequence of the triplet nature of the O_2_ ground state which results in different exchange interactions for the different spin channels. The pristine 3HT (Fig. 1c) shows a singlet ground state indicated by identical spin-up and spin-down DOSs. The lowest unoccupied molecular orbital (LUMO) at −1.09 eV and the highest occupied molecular orbital (HOMO) at −5.55 eV, as well as the HOMO-1 levels, are formed by the atoms of the thiophene ring. More specifically, the atomic orbitals contributing to these levels are the p-orbitals perpendicular to the thiophene plane. Hence, these frontier orbitals are π orbitals. The resulting energy gap was found to be 4.46 eV. The levels which are deeper in energy (<−7 eV) are orbitals localised in the aliphatic side-chain. Due to the stronger bonding of the electrons described by these wave functions, these states are not important for electronic properties. This is the reason why the side-chains are typically neglected in theoretical studies.

So far, we have considered the DOS and pDOS of the individual fragments. Now we turn to the DOS of the complex (Fig. 1d). Comparing the form of the DOS and pDOS of the spin-down density (left panel) to that of the 3HT monomer, no difference can be found. All displayed levels are at similar energies and the contributions of the individual units are nearly identical. The comparison to the spin-down DOS of isolated O_2_ reveals a similar observation with the exception that the two, formerly degenerated levels slightly split because the presence of the 3HT-monomer breaks the symmetry and consequently the degeneration. It can therefore be concluded that the spin-down densities of the two fragments do not interact. On the other hand, the spin-up density reveals a different picture. Here, only the levels deep in energy (<−7 eV) and the LUMO resemble the DOS and pDOS of the isolated 3HT-monomer. The remaining 4 levels which are labelled as L_1_, L_2_, L_3_, and L_4_ can be qualified as follows: L_2_ is mostly localised at the thiophene ring and appears like the pDOS of the 3HT-monomer HOMO. L_3_ on the other hand shows major contribution from the O_2_ molecule indicating equivalence to one of the two π* orbitals, most probably the one with a node plane perpendicular to the thiophene plane. The remaining two levels L_1_ and L_4_ are both constituted by atoms of the thiophene ring and the oxygen molecule indicating hybridised complex orbitals. Due to this, the HOMO energy is slightly higher than that of the pristine 3HT monomer, resulting in an energy gap of 4.20 eV.

The two spin-down states nearly at −3.60 eV shown in Fig. 1d can be understood in a similar fashion as impurities in solid state semiconductors. Due to the lower energy compared to the LUMO level, a photo-excited electron may decay into these states which leads to modified optoelectronic properties. For this reason, these two levels will simply be referred to as trap-states in the following. They can also be filled with electrons directly from the HOMO, either thermally or by photoexcitation, under absorption of a photon in the infrared range. In both cases, the counter-charge of the immobile excited electron is an additional hole in the HOMO. As pointed out above, the HOMO remains barely impacted by the oxygen meaning that the hole remains delocalised and mobile. Hence, a p-doping effect takes place. Since the oxygen is not covalently bound, its removal–for instance caused by thermal induced diffusion–might be possible. The induced modification of the material properties is therefore reversible and explains the experimentally observed reversible degradation.^11–13,15,24,42^

**Fig. 2:**
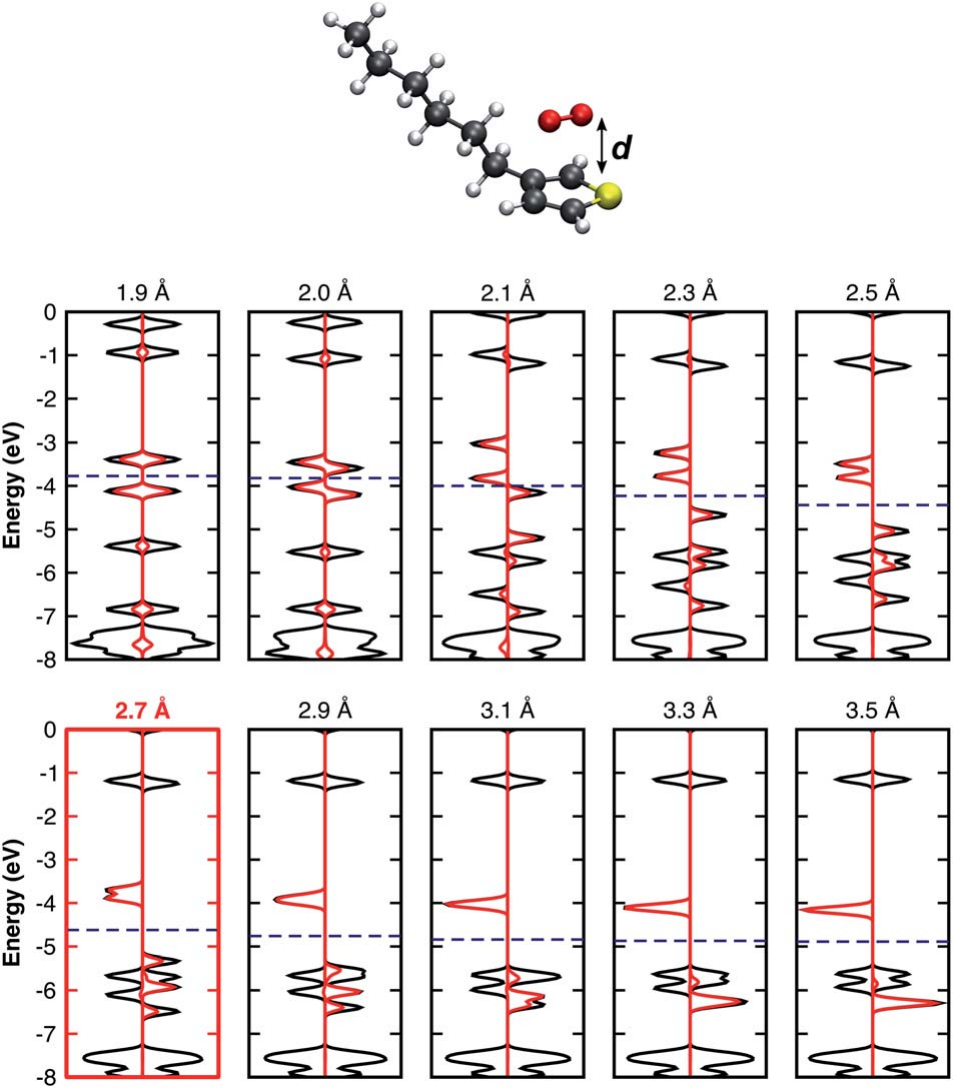
DOS of the 3HT:O_2_ system represented by the solid black lines and the O_2_-pDOS in red, for different single point calculations. Fermi levels are displayed by the blue dashed-lines.

**Fig. 3:**
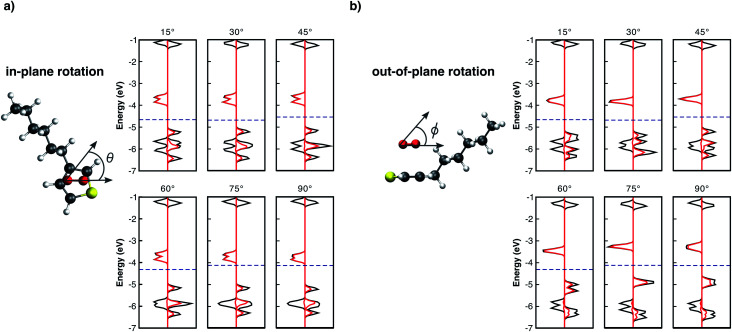
DOS (black lines) of the 3HT:O_2_ system, as well as the pDOS (red lines) of oxygen molecule, for different single point calculations. Fermi levels are displayed by the blue dashed-lines. a) Shows the in-plane angle rotation *θ*, and b) the out-of-plane angle *ϕ*.

It is important to note that all results summarised so far are for isolated complex models surrounded by vacuum. In real organic thin films, complexes are surrounded by other molecules which may lead to different geometrical configurations. The complex geometry in such cases might differ from those obtained from our optimisation of an isolated model. For this reason, we found it important to consider further modified geometries. Thus, we performed single point calculations using the previously relaxed orientation, evaluating the influence of: i) the distance *d* between the 3HT monomer and O_2_ (Fig. 2), ii) in-plane (Fig. 3a), and iii) out-of-plane rotations (Fig. 3b), on the electronic properties. Analysing the pDOS (Fig. 2) one can observe three important aspects when the O_2_ molecule approaches the 3HT monomer (*d* < 2.7 Å). Firstly, by reducing *d*, the physical interaction becomes stronger, resulting in an increased splitting of the two spin-down oxygen trap-states (red line). Secondly, the splitting of the hybridised levels L_1_ and L_4_ of Fig. 2 also increases, shifting the HOMO energy up such that the distance to the lower spin-down trap-state shrinks. Thirdly, for *d* between 2.0–2.1 Å, the splitting is so strong that a change from the triplet to the singlet state occurs. This suggests the first step towards the irreversible degradation as the singlet oxygen is known to be far more reactive to organic compounds, being also responsible for the photo-degradation of many materials. In the opposite case (*d* > 2.7 Å), when the oxygen is moved away from 3HT the splitting of the trap-states as well as of L_1_ and L_4_ decreases, and the two fragments act as isolated systems at a distance of about 3.3 Å.

From Fig. 3a, the in-plane rotation does not promote any significant influence on oxygen trap-states as well as on the monomer HOMO–LUMO states. In addition, while a global energy minimum at *d* = 2.7 Å (Fig. SM1†) was observed, the total energy remaining approximately constant for all in-plane angles. These results are not unexpected because in-plane rotations maintain the p_*z*_ orbitals of the two molecules parallel to each other. Conversely, out-of-plane plane rotations (Fig. 3b) present a different influence on L_1_–L_4_ levels. As the angle *ϕ* increases, L_3_ drastically shifts towards higher energies approaching L_1_. For angles >45°, L_1_ and L_3_ combine and form a new HOMO level. As one can see from Fig. 3b the major contribution of the new HOMO level originates from the oxygen spin-up orbitals. This scenario is similar to the individual molecules because as the angle increases the two initially parallel p_*z*_ orbitals turn perpendicular, thus decreasing the interaction between them. The out-of-plane rotation, however, is energetically not favoured resulting in a decrease of binding energy of up to 0.5 eV (see Fig. SM2†).

### 3-Hexylthiophene oligomer (13-3HT)

It is known that conjugated polymers form amorphous and semicrystalline structures when processed to thin films.^43–45^ In the ordered regions the charge transport is delocalised whereas it is very localised in the amorphous parts. Thin film crystallinity is determined by several parameters and depends on many conditions from synthesis to film processing. The most important ones are: monomer characteristics, chain regioregularity, size and torsions, solvent, the use of additives and others.^20,46–48^ In the attempt of mimicking possible morphological defects, we investigate the effect of oxygen molecules on a regioregular (Rr) and regiorandom (Ra) 13-3HT oligomers in fully planar as well as in twisted orientation. The side and top view of the three models are shown in Fig. SM3.† Fig. SM4† depicts the band diagram for a 3HT-based system, increasing the number of repeat units *N* from 1 to 13. The gap between HOMO and LUMO converges to a value of about 1.15 eV and the energy of the frontier states remains practically the same for *N* > 11. Thus, we consider the 13-3HT as a representation of a P3HT polymer. In the next step, we individually placed the O_2_ molecule on top of each of the 13 repeat units followed by a structure relaxation. For the obtained geometry, the total energy, the DOS, and the pDOS were calculated for all the defect structures.

**Fig. 4:**
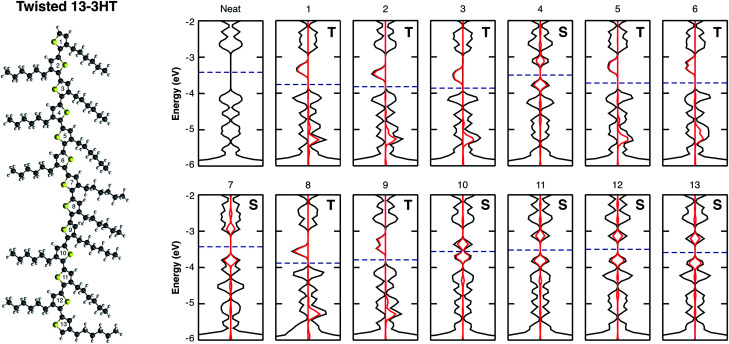
Electronic structure of the neat twisted 13-3HT (first), as well as the 13-3HT:O_2_ complex, in which each band structure corresponds to the site position of the O_2_ molecule with respect to the thiophene ring number (top-to-bottom).

Fig. 4 shows the electronic structure of the twisted 13-3HT oligomer, neat (first) and with the presence of O_2_ (others). Each number corresponds to the position of an individual oxygen molecule with respect to the thiophene ring number, starting from top to the bottom. Differently from the monomer, in which the triplet was the energetically favourable state, stable singlet states were also obtained for calculations of all 13-3HT models. From Fig. 4, however, it is not possible to find a direct correlation between the chain defects and the spin polarity. To better understand the origin of different multiplicities, we analysed the resulting distance between O_2_ and the nearest atom of 13-3HT thiophene ring. We found that singlet states have an average distance of (2.12 ± 0.06) Å, and triplet states of (2.66 ± 0.07) Å. Similar results were observed for Rr and Ra models and the statistics of the spin multiplicity occurrence is depicted in Fig. SM5.†

**Fig. 5:**
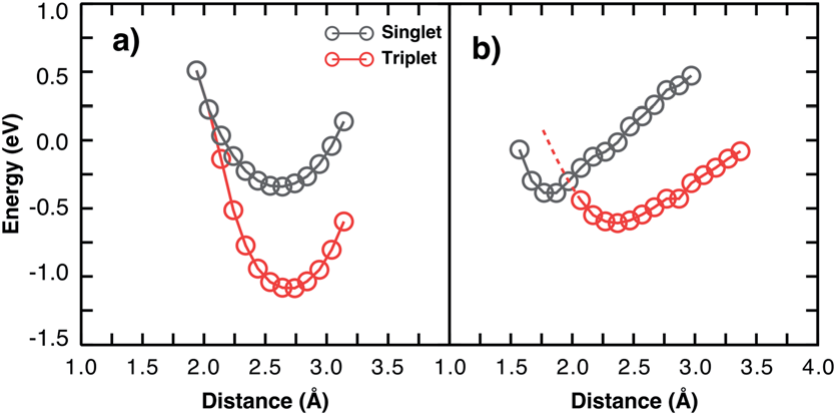
Single point energy calculation for two different thiophene sites (3 and 4) of a twisted 13-3HT oligomer. The energy–distance profile of the two sites exhibits two different types: Type 1, shown in a); and Type 2, in b). Red dashed-line is the extrapolated points for *d* < 2.0 Å.

Considering two particular sites of the 13-3HT oligomer in Fig. 4 we calculated the total energy varying the distance *d* of the O_2_ molecule while keeping its multiplicity fixed. From Fig. 5, the total energy profile calculated on sites 3 and 4 exhibits two different types. For Type 1 (a), the minima of the energy profiles are nearly at the same distance with the triplet state being by about 0.6 eV more stable. This means the triplet state is far more stable and basically only reversible degradation occurs for Type 1. On the other hand, for Type 2 (Fig. 5b), The triplet state is more stable as well, but the local minimum of the singlet state is at smaller distances and below the potential energy surface of the triplet state (red dashed line). This indicates that the singlet state can occur as a meta-stable and is only about 0.26 eV above the minimum of the triplet state. The same observations were made for all 13-3HT oligomers, including the regioregular (Rr), regiorandom (Ra) and twisted but not for the monomer. For the monomer, only a behaviour similar to Type 1 was found (see Fig. SM6†). We therefore conclude that stable singlet states are a feature of polymerized systems. We argue that different thiophene sites promote distinct crystal-fields in their neighbourhood so that the two types are observed. The specific structural aspects of the polymer, however, do not favour or disfavour Type 1 or Type 2. We therefore conclude that irreversible and reversible degradation is not directly promoted by chain defects. Hence, observed correlations between lower structure defect concentration and lower degradation can at most be due to indirect effects such as reduced oxygen diffusion at higher degrees of crystallinity. Hence, the different degradation kinetics resulting from morphological aspects, experimentally observed in disordered films, should be related with the oxygen permeability and not to energetic issues. We also emphasise that, although we restrict our studies to P3HT, we believe that most of our observations and conclusions remain true for other organic compounds and are universal for conjugated systems as polymers and small molecules.

## Conclusions

Here, we have theoretically studied the interaction between a O_2_ molecule with the widely used organic semiconductor polymer P3HT by means of first-principles simulations in the framework of DFT. We observed that upon interaction with molecular oxygen, the 3HT monomer forms a stable complex at a distance of 2.7 Å. Trap-states are introduced by triplet oxygen, leading to the experimentally observed p-doping character. A similar scenario was also observed for different oligomers including chain defects. However, differently from the monomer, singlet oxygen was also obtained as a meta-stable state, irrespectively whether or not chain defects were present. According to our results, the singlet state becomes energetically more favourable than the triplet state for distances below 2.1 Å. This stronger singlet character is the first step towards a chemical interaction *i.e.* an irreversible degradation. We assume that the observed effects here can also be extrapolated to other π-conjugating organic systems and efforts have to be made in order to better protect these materials from oxygen.

## Conflicts of interest

There are no conflicts of interest to declare.

## Supplementary Material
